# Knowledge, attitude, practice and associated factors among patients with type 2 diabetes in Cotonou, Southern Benin

**DOI:** 10.1186/s12889-021-10289-8

**Published:** 2021-02-12

**Authors:** Halimatou Alaofè, Waliou Amoussa Hounkpatin, Francois Djrolo, John Ehiri, Cecilia Rosales

**Affiliations:** 1 Health Promotion Sciences Department, University of Arizona, P.O. Box 245209, 1295 N. Martin Ave., Tucson, AZ 85724 USA; 2 School of Nutrition and Food Science and Technology, Faculty of Agricultural Sciences of the University of Abomey-Calavi (FSA-UAC) Campus d’Abomey-Calavi, 01 BP 526 Calavi, Benin; 3 University of Abomey-Calavi Faculty of Health Sciences, 01 B.P. 188 Champ de foire, Cadjehoun, Cotonou, Benin; 4 Division of Public Health Practice & Translational Research, University of Arizona, 550 E. Van Buren Street, Phoenix Plaza Building, Phoenix, AZ 85006 USA

**Keywords:** Type 2 diabetes, Knowledge, Attitude, Practice, Benin

## Abstract

**Background:**

Type 2 diabetes (T2D) is becoming an epidemic with significant disability and premature death in Sub-Saharan Africa, including Benin. However, little is known about the level of knowledge, attitude, and practice (KAP) necessary for diabetic patients to enhance therapeutic outcomes and prevent diabetes complications. The study aimed to assess patients’ KAP levels and identify the factors associated in Cotonou, southern Benin.

**Methods:**

A cross-sectional study was conducted from July to August 2019 among 300 diabetic patients from four health centers. Data was collected using validated questionnaires. KAP levels were determined by calculating the scores, and multivariate logistic regression was used to explore factors influencing KAP scores.

**Results:**

About 53, 52, and 47% of all patients had good knowledge, attitude, and practice towards diabetes. In logistic regression, factors such as being female, married, educated, government/non-government employee, and longer duration of diabetes were significantly associated with good knowledge. Being married, having a longer duration of diabetes, and good knowledge were significantly associated with a good attitude while being educated, having a longer duration of diabetes, and good knowledge with good practice.

**Conclusions:**

Lack of knowledge, poor attitude, and inadequate practice were found in this surveyed community, suggesting a need for structured educational programs to assist diabetic patients. However, education should be considered a priority for male, newly diagnosed, and uneducated patients.

**Supplementary Information:**

The online version contains supplementary material available at 10.1186/s12889-021-10289-8.

## Background

Type 2 diabetes (T2D), as a significant fast-growing noncommunicable disease (NCD), is a threat to global public health [1]. Sub-Saharan Africa (SSA) is estimated to have 15.9 million adults living with T2D and associated healthcare costs of USD 3.3 billion. By 2045, this number is expected to increase by 162.5%, and costs will rise to USD 6 billion [1]. Due to the current rapid demographic, sociocultural, nutrition, and economic transitions of SSA countries, the increase in diabetes prevalence is expected to beat other global regions [2]. Also, SSA countries face several challenges to address this growing burden, including limited health and social care resources and the continued competing costs of diseases such as HIV/AIDS and malaria [3]. The African continent has the most significant proportion of people with undiagnosed T2D (69%), leading to a high prevalence of diabetes-related complications. In contrast, only 6% of all deaths were attributed to diabetes [1, 4, 5].

Benin – a sub-Saharan country -- is no exception. The prevalence of T2D doubled between 2008 (4.6%) and 2015 (8.4%). The overall diabetes prevalence in some cities of the country reaches 21.6%, with 15.1% in urban and 9.5% in rural areas [6]. T2D is associated with end-stage kidney disease, erectile dysfunction, diabetic foot, stroke, limb amputation, and renal dialysis [7–9]. The diabetes age-standardized death rate was 618 for females and 430 for males, as contrasted to 190.5 and 122 per 100,000 in the US, respectively [10]. Further, the burden of T2D in Benin is more likely to increase with the nutritional transition and accelerating urbanization [11]. Indeed, diabetes was in the top 10 health problems that cause the most disability, with a 55.8% increase between 2007 and 2017. Similar trends were observed for diabetes risk factors that drive the most death and disability combined: high blood pressure (37.7%), dietary risks (30.8%), and high body mass index (43.4%) [12]. Dietary risks include diets low in fruits, vegetables, whole grains, nuts and seeds, fiber, milk, calcium, omega-3 oils, polyunsaturated fatty acids, and high sodium, red meat, and processed meat sweetened beverages, and trans fats [13].

Additionally, Benin has a hybrid healthcare system, inclusive of public and private healthcare service providers. However, most private healthcare providers are concentrated in urban Benin, providing secondary and tertiary care healthcare services [14]. The public healthcare infrastructure has been developed as a three-tier system based on population norms. Patients diagnosed in primary care centers are generally referred to the secondary care centers because they often only have nurses with little experience in diabetes care. There are medical officers and sometimes consultant (specialist) physicians with advanced experience in managing diabetes at the secondary care centers. Other patients who cannot be managed at the secondary health centers because of comorbidities or diabetes complications are referred to tertiary health institutions [14]. Most Beninese with diabetes are seen in secondary institutions (general hospitals, specialist hospitals, and private hospitals). At this tier of care, diabetes educators are scarce, and therefore medical officers often need to assume these roles, while nurses double as diabetes educators.

Knowledge, attitude, and self-care practices (KAP) regarding diabetes are essential for patients to achieve treatment targets and prevent complications [15]. However, only two studies assessed diabetic patients’ knowledge and daily practices in Benin [16, 17]. In Alassani et al.’s study, deficiencies were noted in diabetes knowledge (definition and control of diabetes, symptoms of hypoglycemia) [16]. Difficulties were also encountered in compliance with diet (20%), physical activity (55.7%), and glycemic control (7.8%). In the study by Anvoegbe et al., only 9.1% of patients had good therapeutic adherence [17]. Furthermore, no research has investigated factors associated with diabetes KAP. To fill this knowledge gap, this study assessed the KAP and associated factors among diabetic patients in Cotonou, where the prevalence of T2D is 19.4%, and the disease burden and risk factors are rapidly growing. Understanding these factors is essential in a multicultural society like Benin since different personal and environmental contexts, including demographic, psychosocial, and socioeconomic variables, could influence KAP towards diabetes [16, 18].

## Methods

### Study design, setting, and period

This institution-based cross-sectional study was conducted from July to August 2019 in four secondary care centers of Cotonou: Centre National University Hospital Hubert Maga Koutoucou (CNHU-HKM), Bank of Insulin, and two private clinics (BONI and UNION). CNHU-HKM is the reference center of Benin’s national health system, while Bank of Insulin, created by Lions Clubs, is for screening and managing diabetes. Cotonou is an urban area where T2D risk factors are of equal or higher prevalence than the rest of the country. About 39% of adults (18–69 years) were reported overweight/obese, 93% consumed less than five servings of fruits and vegetables (F&V) per day, 83% were physically inactive, and 32% were diagnosed with hypertension. Between 2008 and 2015, diabetes prevalence increased from 4 to 19%, while overweight/obesity among women doubled (23 to 50%) [6].

### Study population and eligibility criteria

Type 2 diabetes patients receiving diabetes care at the outpatient department of the diabetes unit in the four health centers were included in the study except for those in an extreme disease condition that restricts them from responding to questionnaires. Specifically, the participants were sampled and included in the study if they met the following inclusion criteria: (1) aged 18 years and above; (2) have been living with T2D for a year or more; (3) willing to give informed consent to participate in the study.

### Sample calculation and recruitment

The sample size was estimated by considering a single population proportion formula assuming a 95% confidence interval, 1.96 standard normal variables (z score) with a 5% margin of error. Accordingly, the sample size became 238. By adjusting the prevalence of diabetes in Cotonou to 19.2, and 10% contingency, it became 263 patients. In total, 300 T2D patients were recruited using a disproportionate stratified sampling method to ensure representativeness between centers. Accordingly, we selected 38 patients from CNHU-HKM, 163 from the Bank of Insulin, 50 from the clinic BONI, and 49 from the clinic UNION.

This study’s proposal was sent to the four institutions’ head to seek permission to use the center as the study site. After the facility’s authorization, potential participants were randomly selected from the list of T2D patients on the diabetic registration chart (with their respective medical registration number) using a computer-generated table of random numbers. Selected patients were approached, the study’s objectives were explained, and those who agreed to participate in the study gave consent.

### Data collection instrument and procedure

The data was collected in French or local languages (when needed) using a pretested interviewer-administered questionnaire (Patient KAP questionnaire). The questionnaire was adapted from relevant literature [19–21] and translated to French, followed by back translation to English, to ensure the data collection tool’s validity. Four enumerators holding Master of Science degrees in Nutrition at the University of Abomey Calavi were involved in data collection under the principal investigator’s supervision (PI).

The questionnaire had four sections. In the first section, socio-demographic and clinical data were collected on age, sex, religion, level of education, marital status, occupation, duration of diabetes, family history of T2D, comorbidities, body mass index (BMI), and having an insurance plan. The diagnosis of comorbidities such as nephropathy, retinopathy, and neuropathy was made through the questionnaire and confirmed on their diabetic registration charts or clinical records. The second section included knowledge questions (*n*=11) developed from an existing validated questionnaire in the ‘Spoken Knowledge in Low Literacy in Diabetes knowledge assessment Scale’ (SKILLDs) [19]. The third section was focused on attitude questions (*n*=16) developed from the Diabetes Attitude Survey (DAS3) of the University of Michigan Diabetes Research and Training Center [20]. The last section included self-care practice questions (*n*=10) adapted from the Diabetes KAP developed for Iranian patients with type 2 diabetes [21].

Specifically, knowledge questions were multiple-choice questions with 0–1 and 0–4 scores, based on the correct choices. Questions on attitude were − 2 to + 2 on a Likert scale: strongly agree, agree, no idea, disagree, and strongly disagree. Each question in the practice section earned 1 point for correct practice and 0 points for incorrect practice. The total score ranges were 0–26 for the knowledge section, − 32 to + 32 for the attitude section, and 0–10 for the practice section. Mean scores of diabetes KAP were calculated and used to classify the respondents into two groups. Good diabetes knowledge was classified as a mean score in the SKILLDs equal to or higher than the mean average. Similarly, participants who scored equal to or above the mean score in the DAS3 were classified as having a good attitude. Finally, a good self-care practice was indicated by a mean score in the Iranian practice questionnaire equal to or higher than the mean average.

### Data quality management/control

Data collectors were given one-day training by the principal investigator (PI) about the data collection procedure and ethical issues. The pretest was conducted on 20 T2D patients in another hospital to assess the content suitability and flow. The collected data were checked for completeness at a daily meeting.

### Statistical analysis

Data were entered and cleaned using Redcap hosted at the University of Arizona [22, 23] and then transported to Stata version 14 for analysis. Frequency distributions were computed for demographic variables, and mean values with standard deviations (± SD) were calculated for both the individual dimensions of the DAS3 and overall diabetes knowledge and self-care practice scales. Four assumptions of logistic regressions were checked using STATA codes: independence of observations using the Durbin-Watson statistic, multicollinearity through the inspection of Tolerance/VIF values, linear relationship, and no significant outliers using scatterplots and regression plots. Then, bivariate analysis was done for every single independent variable (age, sex, religion, level of education, marital status, occupation, duration of diabetes, family history of T2D, comorbidities, BMI, and having an insurance plan) with levels of KAP (“good” vs. “poor”). Factors with a *p*-value of 0.2 and below were entered into a multivariable logistic regression model to recognize KAP’s independent associated factors. KAP levels were also included in the multiple logistic regression to explore the relation between KAP domains. Adjusted odds ratio (AOR) with 95% CI and the p-value < 0.05 were considered to declare significantly associated factors.

## Results

### Participants’ demographic and clinical characteristics

As shown in Table 1, from the total 300 patients, the majority were female (70.7%), Christian (79.3%), married (59.3%), and had a family history of diabetes (61%). Most respondents also represented the age group 40–59 years (56.6%), and 38.7% were above 60 years. One-fifth (18%) of them had no formal education. About half of the respondents were self-employed (52.3%). The mean duration of the disease was 8.2 years, and only 10% had insurance coverage. About 49% were overweight/obese, while 34.7, 46.7, and 51.7% had retinopathy, neuropathy, and hypertension, respectively.

**Table 1:** Demographic and clinical characteristics of the study participants (*N*=300)

Variables	Number	Percent
**Age (mean ± SD), years**	54.93±11.35	
≤39 years	26	8.67
40–59 years	158	**52.67**
≥ 60 years	116	**38.67**
**Sex**		
Male	88	29.33
Female	212	**70.67**
**Religion**		
Christian	238	**79.33**
Muslim	46	15.33
Others^a^	16	5.33
**Education**		
No formal education	55	**18.33**
Primary school	94	**31.33**
Secondary school	97	**32.33**
College & above	30	10.00
**Marital status**		
Single	48	16.00
Married	178	**59.33**
Separated/Divorced	9	3.00
Widowed	65	21.67
**Occupation**		
Government employee	22	7.33
Non-government employee	17	5.67
Self-employed	157	**52.33**
Retired	40	13.33
Others^b^	64	21.33
**Duration of Diabetes (mean ± SD), years**	8.18±8.58	
**Family history of DM**	183	**61.00**
**Comorbidities**		
Hypertension	155	**51.67**
Dyslipidemia	19	6.33
Nephropathy	21	7.00
Retinopathy	140	**46.67**
Neuropathy	104	**34.67**
**BMI (mean ± SD), kg/m**^**2**^	29.29±6.75	
Underweight	3	1.00
Normal	42	14.00
Overweight	62	**20.67**
Obese	84	**28.00**
Don’t know	109	36.33
Have assurance plan	30	**10.00**

mean ± SD = mean ± Standard Deviation

^a^ Others=Eckist and Vodoun; ^b^ Student, Housewife, Unemployed

### Participants’ diabetes-related knowledge

From Table 2, participants responded correctly to questions affirming that diabetes is incurable (93%), defined as high levels of sugar in the blood (91%), and affects any part of the body (69%). Participants stated that overweight/obesity (60.3%), family history (60.3%), and poor dietary habits (55.6%) could predispose them to develop diabetes. Regarding signs and symptoms of diabetes, excessive thirst (48.3%) and high blood sugar (48.3%) were highly rated. They also indicated that diabetes could be controlled by medication (89.7%) and the practice of a healthy diet (64.7%). Kidney failure, eye problems, and amputation of limbs were significant complications of diabetes identified (> 87%).

**Table 2:** Distribution of participants’ diabetes knowledge response

Knowledge	Mean	n	%
**What is diabetes? (0–4)**			
Diabetes is a condition of insufficient insulin production	0.08±0.27	23	7.67
Diabetes is a condition of high level of sugar in the blood	0.91±0.29	273	91.00
Diabetes is not curable	0.93±0.26	279	93.00
Diabetes is a disease that affect any part of body	0.69±0.46	207	69.00
**Which one could cause type-2 diabetes? (0–4)**			
Genetic or family history	0.61±0.49	183	61.00
Being overweight/obese	0.71±0.46	212	70.67
Sedentary life	0.47±0.50	142	47.33
Poor dietary habits	0.61±0.49	182	60.67
**What are diabetes symptoms? (0–4)**			
Excessive thirst	0.48±0.50	145	48.33
Excessive hunger	0.19±0.40	58	19.33
High blood sugar	0.48±0.50	145	48.33
Feeling of weakness	0.19±0.40	58	19.33
**What is necessary for controlling diabetes? (0–4)**			
Medication	0.90±0.30	269	89.67
Regular exercise	0.47±0.50	142	47.33
Practice healthy diet	0.61±0.49	182	60.67
Medical eye/foot checkup or care	0.49±0.50	148	49.33
**What are diabetes complications? (0–4)**			
Diabetes can cause eye problem or even blindness	0.89±0.31	267	89.00
Diabetes can cause kidney failure	0.92±0.27	276	92.00
Diabetes can cause heart failure	0.46±0.50	138	46.00
Diabetes can result in amputation of limb	0.87±0.33	262	87.33
**What is the effect of exercise on glucose controlling? (0–1)**	0.83±0.37	250	83.33
**Is dietary intervention necessary in controlling glucose? (0–1)**	0.75±0.44	224	74.67
**Which one is the normal blood glucose in a healthy person? (0–1)**	0.65±0.48	194	64.67
**What is suitable blood pressure for a diabetic patient? (0–1)**	0.50±0.50	151	50.33
**Which index is used to get an average blood sugar reading? (0–1)**	0.18±0.38	54	18.00
**Which one is the correct foot care in a diabetic person? (0–1)**	0.64±0.48	193	64.33

Mean (±SD) sub score for diabetes definitions (out of 4 total points)= 2.61±0.69

Mean (±SD) sub score for diabetes risk factorsT2DM (out of 4 total points)=2.40±1.31

Mean (±SD) sub score for diabetes symptoms (out of 4 total points)= 1.35±1.41

Mean (±SD) sub score for control of diabetes (out of 4 total points)= 2.47±0.98

Mean (±SD) sub score for complications of diabetes (out of 4 total points)=3.14±0.87

Total mean (±SD) Knowledge score (out of 26 total points)= 15.52±3.34

% of patients ≥mean=52.67

*SD* Standard Deviation

When asked about blood sugar levels, 64.7% of participants knew the expected value of 70–110 mg/dl. Around 64% of them knew the correct foot care in a diabetic person. Most of them correctly stated the effect of exercise (83.3%) and diet (74.7%) on glucose control. However, only half knew suitable blood pressure for a diabetic patient. Participants also had a low frequency of correct responses on the diabetes definition due to insufficient insulin production (7.7%) and the index suitable for awareness about diabetes control in past months (19.3%). They had a frequency below half in diabetes symptoms, risk factors, and control, as they did not relate regular exercise, sedentary life, and heart failure with diabetes. The total mean score for knowledge questions was 15.52±3.34 (out of 26 total points). About 53% scored at or above the mean and were considered knowledgeable.

### Participants’ diabetes-related attitude

In Table 3, the mean score of the participants’ attitude was 11.24±6.7 (out of 32 points). About 47.7% of participants scored below the mean score (considered poor attitude) and 52.3% at or above the mean (considered good attitude). The majority of the respondents strongly stated that diabetes has a significant negative impact on the patient’s life (4.74±3.65 out of 10 total points). Most also believed that health care professionals need specialized training to care for persons with diabetes (2.98±1.15 out of 4 total points) and were supportive of patients being in charge of their diabetes management (2.72±1.44 out of 4 total points). However, there were vast differences in opinion among the respondents for diabetes as a severe disease (1.15±2.88 out of 8 total points) and the fact that reasonable blood glucose control reduces the likelihood that complications will develop (− 0.36±2.35 out of 6 total points). Only 27.7 and 39.4% disagreed and strongly disagreed that people who do not need to take insulin to treat their diabetes have a mild disease, and people whose diabetes is treated by just a diet do not have to worry about getting many long-term complications, respectively. Similarly, 25.7 and 27.3% disagreed or strongly disagreed that low blood sugar reactions make tight control riskier for most people, and tight control is too much work, respectively.

**Table 3:** Distribution of participants’ diabetes attitude response

Attitude	Mean	Strongly agree	Agree	Neutral	Disagree	Strongly disagree
	X±SD	n(%)	n(%)	n(%)	n(%)	n(%)
**Need for special training**						
Health care professionals who treat people with diabetes should be trained to communicate well with their patients	1.70±0.56	221 (73.67)	70 (23.33)	6 (2.00)	3 (1.00)	0
Health care professionals should learn how to set goals with patients, not just tell them what to do.	1.29±0.86	151 (50.33)	99 (33.00)	36 (12.00)	13 (4.33)	1 (0.33)
**Seriousness of T2D**						
People who do not need to take insulin to treat their diabetes have a mild disease.	−0.42±1.20	69 (23.00)	85 (28.33)	63 (21.00)	69 (23.00)	14 (4.67)
People whose diabetes is treated by just a diet do not have to worry about getting many long-term complications.	−0.10±1.28	50 (16.67)	80 (26.67)	52 (17.33)	86 (28.67)	32 (10.67)
Blood sugar testing is not needed for people with T2D	0.77±1.08	14 (4.67)	34 (11.33)	31 (10.33)	149 (49.67)	72 (24.00)
People who take diabetes medications should be as concerned about their blood sugar as people who take insulin.	0.90±1.07	104 (34.67)	110 (36.67)	40 (13.33)	43 (14.33)	3 (1.00)
**Value of tight control**						
There is not much use in trying to have good blood sugar control because the complications of diabetes will happen anyway.	0.26±1.34	43 (14.33)	60 (20.00)	26 (8.67)	119 (39.67)	52 (17.33)
Low blood sugar reactions make tight control too risky for most people	−0.14±0.93	26 (8.67)	70 (23.33)	127 (42.33)	73 (24.33)	4 (1.33)
Tight control is too much work	−0.48±1.22	78 (26.00)	82 (27.33)	58 (19.33)	69 (23.00)	13 (4.33)
**Psychological impact of T2D**						
Diabetes affects almost every part of a diabetic person’s life.	0.67±1.13	74 (24.67)	128 (42.67)	33 (11.00)	56 (18.67)	9 (3.00)
The emotional effects of diabetes are small.	0.04±0.96	16 (5.33)	69 (23.00)	117 (39.00)	82 (27.33)	16 (5.33)
Diabetes is hard because you never get a break from it.	0.90±1.10	101 (33.67)	123 (41.00)	29 (9.67)	38 (12.67)	9 (3.00)
Having diabetes changes a person’s outlook on life.	0.86±1.12	101 (33.67)	116 (38.67)	34 (11.34)	39 (13.00)	10 (3.33)
Support from family and friends is important in dealing with diabetes.	1.59±0.70	208 (69.33)	70 (23.33)	14 (4.67)	8 (2.67)	0
**Patient autonomy**						
People with diabetes should learn a lot about the disease so that they can be in charge of their own diabetes care.	1.50±0.84	205 (68.33)	52 (17.33)	32 (10.67)	10 (3.33)	1 (0.33)
What the patient does has more effect on the outcome of diabetes care than anything a health professional does.	1.22±0.88	138 (46.00)	108 (36.00)	39 (13.00)	13 (4.33)	2 (0.67)

Mean (±SD) sub score for need for special train (out of 4 total **points)= 2.98±1.15**

Mean (±SD) sub score for seriousness of T2DM (out of 8 total points)= 1.15±2.88

Mean (±SD) sub score for value of tight control (out of 6 total points)= −0.36±2.35

Mean (±SD) sub score for psychological impact of T2DM (out of 10 total points**)=4.74±3.65**

Mean (±SD) sub score for patient autonomy (out of 4 total points)=**2.72±1.44**

Total mean (±SD) attitude score (out of 32 total points)= 11.24±6.72

% of patients ≥mean=52.33

### Participants’ diabetes-related practice

In Table 4, the majority of participants reported to have three main meals daily (85.7%) and did not smoke or drink alcohol (75%). However, less than 30% of them checked their eyes yearly (18%), had glucometers (20.3%), had ever participated in a diabetes education class (24.3%), examined their feet daily (27.7%), and had a meal plan (29.7%). Only 35.3% of the patients admitted to regular exercise, and just 42% visited the doctor three times a year. The mean score of the participants’ practice level was 3.80±1.59 (out of 10 total points). One hundred sixty participants scored below the mean (53.3%), which was considered poor practice, and 140 scored at or above the mean (46.7%), which was considered good practice.

**Table 4:** Distribution of participants’ diabetes practice response

Practice	Mean	n	%
When was your last eye exam?	0.18±0.38	54	18.00
Do you have a meal plan?	0.30±0.46	89	29.67
How many times a week do you examine your feet?	0.28±0.45	83	27.67
Have you glucometer?	0.20±0.40	61	20.33
Do you test your blood sugar?	0.52±0.50	155	52.01
How many days a week do you exercise?	0.35±0.48	106	35.33
How many main meals do you have daily?	0.86±0.35	257	85.67
Last year, how many times did you visit a doctor?	0.58±0.49	126	42.00
Do you smoke or drink alcohol?	0.75±0.43	205	75.09
Have you ever participated in a diabetes education class?	0.24±0.43	73	24.33

Total mean (±SD) practice score (out of 10 total points)= 3.80±1.59

% of patients ≥mean=46.67

### Factors associated with participants’ KAP

In the multivariate logistic analysis in Table 5, female, educated, married, and government/non-government employed patients were respectively 1.91 (95%CI = 1.9–3.8), 1.54 (95%CI = 1.1–2.3), 2.64 (95%CI = 1.4–4.9), and 2.21 (95%CI = 1.8–5.9) times more likely to have good diabetes knowledge as compared to male, not educated, single/separated/widowed and unemployed/self-employed patients. Similarly, patients with a duration of disease ≥10 years were 4.28 times (95%CI = 2.1–9.7) more likely to have good diabetes knowledge than those with < 10 years.

**Table 5:** Multivariable analysis of factors associated with KAP towards diabetes

Variables	Knowledge		Attitude		Practice	
	AOR	95%CI	AOR	95%CI	AOR	95%CI
**Age (mean ± SD), years**						
20–39 years	Reference					
≥40 years	1.29	0.48–3.44				
**Sex**						
Male	Reference				Reference	
Female	1.91	**1.95–3.84**			1.44	0.73–2.82
**Religion**						
Christian	Reference		Reference		Reference	
Muslim	1.18	0.54–2.55	1.44	0.71–2.91	1.66	0.78–3.51
**Education**						
No formal education	Reference		Reference		Reference	
Educated	1.54	**1.06–2.32**	1.05	0.79–1.41	3.27	1.23–8.70
**Marital status**						
Not married	Reference		Reference		Reference	
Married	2.64	**1.43–4.88**	1.60	1.94–2.72	1.48	0.82–2.69
**Occupation**						
Others	Reference		Reference		Reference	
Government/non-government employee	2.21	**1.82–5.97**	1.54	0.62–3.85	2.53	1.86–4.70
Self-employed	1.72	0.93–3.19	0.94	0.54–1.64	1.16	0.86–1.56
**Duration of Diabetes**						
< 10	Reference		Reference		Reference	
≥10	4.28	**2.11–9.66**	1.87	1.68–3.02	2.43	1.93–6.34
**Family history of DM**						
No	Reference		Reference			
Yes	1.05	0.59–1.87	0.85	0.49–1.45		
**Diabetes Knowledge**						
Poor			Reference		Reference	
Good			1.69	1.01–2.88	1.78	1.02–3.09
	*N*=245		*N*=292		N=245	
	R^2^=35.95		R^2^=24.58		R^2^=21.46	

*AOR* Adjusted Odds Ratio; 95% CI=95% Confidence Interval

As for attitude levels towards diabetes, married patients were 1.6 times (95%CI = 1.9–2.7) more likely to have a positive attitude than single/separated/widowed patients. Patients with a duration of ≥10 years were 1.87 times (95%CI = 1.7–3.0) more likely to have a good attitude than those with a duration < 10 years. Regarding diabetes knowledge level, patients with good knowledge were 1.6 times (95%CI = 1.1–2.9) more likely to have a positive attitude than those with poor knowledge.

Finally, for diabetes practice levels, educated and government/non-government employee patients were 3.27 (95% CI = 1.2–8.7) and 2.53 (95% CI = 1.9–4.7) times more likely to practice than those without education and unemployed/self-employed. Patients with a duration of ≥10 years were 2.43 times (95%CI = 1.9–6.3) more likely to have good practice than those with a duration of < 10 years. T2D patients who had good diabetes knowledge were 1.78 times (95% CI = 1.02–3.1) more likely to have good practice regarding diabetes than those with poor knowledge.

## Discussion

Diabetes is a chronic disease with different complications requiring broad knowledge and management [24]. Unfortunately, like some studies in Africa [25] and the Middle East [26, 27], this study reveals poor diabetes knowledge, suggesting a need for interventions to improve understanding of the condition among diabetic patients. However, past work with mixed results exists, with some studies reporting lower results similar to ours [1, 28], but others reported higher levels of diabetes knowledge among patients with diabetes [29, 30]. It is difficult to compare our results with others, as these studies used different instruments or were carried out among different ethnic or age groups. Specifically, in this study, patients could generally define diabetes and indicate its risk factors, signs, and symptoms, as well as strategies to control the disease and its complications, as previously observed in Cotonou [16]. However, a shallow frequency was noted on diabetes as a condition of insufficient insulin production, hypoglycemia symptoms, healthy blood sugar level, the importance of hemoglobin A1C, and correct foot care, as well as the relationship between physical activity, heart failure, and diabetes. These findings highlight the need to improve the quality of care that can ultimately result in reduced morbidity.

In diabetes attitude, we found a poor level of attitude compared to studies from South India and the United Arab Emirates, which reported a more positive attitude among patients with diabetes [31, 32]. Our results were similar to Anderson et al.’s study in the US [33], where most respondents firmly stated that diabetes had a significant negative influence on their life. This finding suggests that diabetes and its complications detract significantly from most patients’ quality of life in the surveyed community. Most respondents in our study agreed that health care professionals should have specialized training to care for diabetic patients, as recommended by Pastakia et al. [34]. They were also supportive of patients being in charge of their diabetes management, consistent with the doctor-patient relationship’s traditional view [35]. However, this belief may be counterproductive in the surveyed community as the management of diabetes requires daily self-management behaviors (e.g., self-monitoring of blood glucose) and frequent decisions about medication, diet, and activity levels. Indeed, most patients believe they should be in charge of their diabetes management, but they are not actually managing it well independently, and they go to the doctor infrequently, so they do not realize it or get help with it.

Surprisingly, most of the respondents did not view diabetes as a severe disease, probably due to their belief that people who do not take insulin to treat their diabetes have mild disease. Another possible explanation is the perception of diabetes patients who attributed diabetes to witchcraft or bewitchment in Cotonou [16]. Patients’ belief about the severity of diabetes has important implications for patient education because diabetes control requires long-lasting and challenging behavior changes. Such changes are unlikely sustainable unless patients understand and accept the severe nature of the disease. It will be crucial for health care professionals to strike a balance between false reassurance of insulin-dependent diabetes and unnecessary fear when discussing noninsulin-dependent diabetes with patients. Moreover, most respondents did not believe in the relationship between blood glucose control and complications, although most diabetic patients are encouraged to control their blood glucose to prevent the complications of diabetes. One possible explanation is the fatalistic attitude observed by Al-Sahouri et al. [36]. Because of the multiple possibilities for patients to misunderstand their condition’s seriousness, a holistic approach to diabetes management is needed to assist patients in the region [37].

Although behavior changes and intensive lifestyle interventions are critical components in the management of T2D, the mean score of T2D-related practices was low. This score is part of a more complex picture, however. In our study, 47 and 61% of participants were knowledgeable about the benefits of exercise and a healthy diet; however, only 30% reported having a meal plan and exercising every day, suggesting that efforts should close the gap between knowledge and practice. In that same vein, we observed that less than 30% of patients checked their eyes yearly, examined their feet daily, had their blood sugar checked, and 42% visited a doctor. Similar results were found by Alassani et al. in Cotonou, where difficulties encountered among patients with diabetes were related to physical activity, diet, and glycemic control and observed respectively at 56, 20, and 8% of patients [16]. The reasons given were laziness, shame, fatigue for physical activity, hunger for the diet, and lack of financial means and distance between the residence and the laboratory for glycemic control. This finding suggests complementary education with other interventions such as programs that help coordinate transportation to the laboratory or send phlebotomists into the community.

Some participants’ destructive behaviors and demographic factors associated with increased diabetes-related distress were also observed in the present study. Indeed, control of obesity is essential for better glycemic control and prevention of complications, but it is evident in this study that diabetic subjects do not attain this ideal goal, as 49% are overweight/obese. This finding is in line with studies in Sudan [38] and Tanzania [39]. Another factor of concern is the wrong perception of the majority of the patients to assess their weight. Participants were asked to evaluate their body size, and 64% of overweight patients do not consider themselves to be in that group. Our study also revealed that most patients were diagnosed with T2D after 40, as previously observed in low- and middle-income countries [1], suggesting a need to start screening at an earlier age. Finally, the increased burden of T2D in the region could be explained by the high prevalence of risk factors observed in the present study, such as physical inactivity, smoking, harmful alcohol use, and food habits [11]. There is thus an urgent need to improve education strategies to prevent or delay diabetes-related consequences and hazards.

Similar to studies in Ethiopia [15, 25] and Dhaka [27], our study found significant associations between diabetes-related knowledge with education levels, marital status, occupation, and duration of the disease. However, contrary to our study, males have a better knowledge of the disease than females in India [40] and Zimbabwe [41]. We also found that marital status, duration of diabetes, and knowledge towards the disease were significantly associated with attitude, as observed in Ethiopia [15, 29] and South Africa [42]. Finally, similar to studies in Ethiopia [15, 29] and South Africa [42], education, duration of diseases, and diabetes knowledge diabetes showed a significant association with practice. Our findings also indicated that diabetes knowledge had the highest percentage score, followed by attitude and then practice, suggesting that diabetes knowledge and attitude among patients with diabetes are not reflected in their daily practice, as previously observed in Jordan [31]. However, the fact that good knowledge was significantly associated with attitude and practice indicates that interventions aimed at improving knowledge could benefit patients more than one way.

To our knowledge, this study is the first to assess KAP levels and associated factors among patients with diabetes seen in Benin’s four health centers. However, there are some limitations. This study was restricted to one geographic region and cannot generalize our results to all patients with diabetes in Benin. This study was a cross-sectional survey; therefore, only associations can be determined and not causations. Socioeconomic status or income is a social determinant of disease outcome, but we could not use it in our study because of incomplete or missing data. Data of the two sexes reported were not analyzed separately because of the significant difference in the percentage of female and male participants in the study. Self-reported questionnaires were used for most of the reported measures. Thus, bias may be present due to inaccurate self-reporting, misunderstanding of the questionnaire items, or social desirability. Finally, the study was conducted on type 2 diabetic patients without considering their diabetic complication history status during the data collection period, which positively or negatively affects their attitude and practice level. However, patients who were severely ill were excluded.

## Conclusions

This study revealed poor knowledge, a negative attitude, and poor practices related to diabetes among patients with diabetes in Cotonou, Benin. Therefore, effective health education interventions are needed to improve diabetes knowledge, attitude, and practices, particularly concerning lifestyle modifications and dietary management, to slow down the progression of diabetes and prevent downstream complications. However, interventions related to diabetes and its risk factors should target specific groups of the population, particularly men and those with lower education, not married, and newly diagnosed patients, to ensure benefits from individual, societal, and health-economic perspectives.

## Supplementary Information

**Additional file 1.**

## Data Availability

The datasets used and/or analyzed during the current study are available from the corresponding author on reasonable request.

## Abbreviations

T2D: Type 2 diabetes mellitus
KAP: Knowledge, attitude and practice
NCD: Noncommunicable disease
SSA: Sub-Saharan Africa
CNHU-HKM: Centre National University Hospital Hubert Maga Koutoucou
F&V: Fruits and vegetables
BMI: Body mass index (BMI)
SKILLDS: Spoken knowledge in low literacy in diabetes knowledge assessment scale
DAS 3: Diabetes Attitude Survey 3
