# Supplementary material for: Knowledge, attitude, practice and associated factors among patients with type 2 diabetes in Cotonou, Southern Benin
Source: BMC Public Health. 2021 Feb 12;21:339. doi: 10.1186/s12889-021-10289-8 (PMC7881446; doi:10.1186/s12889-021-10289-8)
Supplement: Supplementary file 1 — Additional file 1. [file 12889_2021_10289_MOESM1_ESM.doc]

**Formative research to inform the adaptation of Meta Salud Diabetes in Benin**

**Diabetes knowledge, attitudes, and self-management**

**UNIVERSITY OF ARIZONA**

**I. Socio-demographics**

**General Information**

1. Centre ID: _____________________Centre name: __________________________________

2. Interviewer ID: _______________________________________________________________

3. Date of completion: ___________________________________________________________

4. Interview language: _1_ French _2_Other____________________________________________

5. Name: ______________________________________________________________________

6. Sex: _1_Male _2_Female

7. Age (years): _________________________ Birth date: __ __ /__ __ /__ __

(Month/Day/Year )

8. Contact Phone (if possible): ____________________________________________________

9. Primary/diabetes physician: _1_No _2_Yes, name ____________________________________

10. What is your race or ethnic background? _1_Fon _2_Mina _3_Refused _4_Other: _____________

**Socioeconomic/ Support System**

1. Marital status: _1_Never married _2_Currently married _3_Separated _4_Divorced _5_Widowed _6_Cohabitating

2. Occupation: _1_Government employee _1_Non-government employee _1_Self-employed _2_Non-paid _3_Student _4_Homemaker _5_Retired _6_Unemployed (able to work) _7_Unemployed (unable to work)

3. Last grade of school completed: _1_No formal schooling _2_Less than primary school _3_Primary school completed _4_Secondary school completed _5_High school completed _6_College/University completed _7_Post-graduate degree

4. Religion? _1_Chritianism _2_Muslim _3_Other_______________________________________

5. Do you have an assurance plan _1_No _1_Yes (Which one?): ___________________________

6. Taking the past year, what the average earnings of the household have been? ______________

**Cultural valuation of body size**

1. Height: ________ Weight: ________
2. Has your weight changed in the past three months? _1_No _2_Yes
3. Which figure is most like you?
4. Which figure would you most like to be in the future?
5. Which male figure appears most healthy?
6. Which female figure appears most healthy?
7. Which male figure appears most attractive/beautiful (just right)?
8.
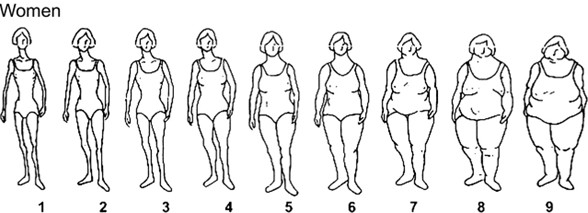
Which female figure appears most attractive/beautiful (just right)?

**
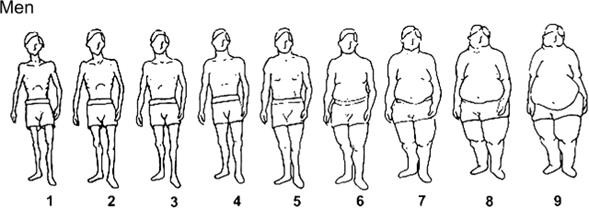
**

**II. Diabetes History**

1. What type of diabetes do you have? _1_Type 1 _2_Type 2 _3_Gestational _4_Don’t know
2. How long have you had diabetes or year diagnosed? _________________________________

**Family History**

1. List any family members with diabetes: ____________________________________________

With high blood pressure: _______________________________________________________

With heart attacks or other heart problems: _________________________________________

With cancer: _____________________________________

**Medical History**

1. Have you ever been diagnosed, ever been told, or have you had problems with the following?

_1_High Blood pressure _2_High Cholesterol/Triglycerides _3_Kidney/Bladder problems _4_Eye or vision problems _5_Heart disease/Chest pain _6_Thyroid disease _7_Numbness/nerve pain/tingling of hands/feet _8_Depression or anxiety _9_Stroke _10_Circulation problems _11_Shortness of Breath _12_Other health problems:_____________

1. **Diabetes Knowledge**
2. In your own word, what is diabetes?
3. Diabetes is a condition of insufficient insulin production
4. Diabetes is a condition of high level of sugar in the blood
5. Diabetes is not curable
6. Diabetes is a disease that affect any part of body
7. Which one could cause diabetes?
8. Genetic or family history
9. Being overweight/obese
10. Sedentary life
11. Poor dietary habits
12. What are diabetes symptoms?
13. Excessive thirst
14. Excessive hunger
15. High blood sugar
16. Feeling of weakness
17. What is necessary for controlling diabetes?
18. Medication
19. Regular exercise
20. Practice healthy diet
21. Medical eye/foot checkup or care
22. What are some long-term complications of uncontrolled diabetes?
23. Diabetes can cause eye problem or even blindness
24. Diabetes can cause kidney failure
25. Diabetes can cause heart failure
26. Diabetes can result in amputation of limb
27. What is the effect of exercise on the glucose controlling?
28. Lowers it
29. Raises it
30. Has no effect
31. Is dietary intervention necessary in controlling glucose? 1. No 2. Yes
32. What is a normal fasting blood glucose or blood sugar? _______________________________
33. What is a normal blood pressure for a diabetic patient? ________________________________
34. Which index is used to get an average blood sugar reading? ____________________________
35. Which one is the correct foot care in a diabetic person?
36. Look at and wash them each day
37. Massage them with alcohol each day
38. Soak them for one hour each day
39. Buy shoes a size larger than usual
40. **Diabetes Attitude**

Below are some statements about diabetes. Each numbered statement finishes the sentence “In general, I believe that...” Please, give the answer that you believe is true most of the time or is true for most people.

Note: The term “health care professionals” in this survey refers to doctors, nurses, and dietitians.

**Strongly Strongly**

**Agree Agree Neutral Disagree Disagree**

**In general, I believe that**

1. ...health care professionals who

treat people with diabetes should

be trained to communicate well

with their patients.

2. ...health care professionals should

learn how to set goals with patients,

not just tell them what to do.

1. ...people who do not need to take

insulin to treat their diabetes have

a mild disease.

1. ...people whose diabetes is treated

by just a diet do not have to worry

about getting many long-term

complications.

5. ...blood sugar testing is not needed

for people with Type 2 diabetes.

6. ...people who take diabetes pills

should be as concerned about their

blood sugar as people who take insulin.

7. ...there is not much use in trying to

have good blood sugar control

because the complications of

diabetes will happen anyway.

8. ...low blood sugar reactions make

tight control too risky for most

people.

9. ...tight control is too much work.

10. ...diabetes affects almost every

part of a diabetic person’s life.

11. ...the emotional effects of diabetes

are pretty small.

12. ...diabetes is hard because you

never get a break from it.

13. ...having diabetes changes a

person’s outlook on life.

14. ...support from family and friends

is important in dealing with

diabetes.

15. ...people with diabetes should

learn a lot about the disease so that

they can be in charge of their own

diabetes care.

16. ...what the patient does has more

effect on the outcome of diabetes

care than anything a health

professional does.

1. **Diabetes Self-care Practice**
2. When was your last eye exam? How often did you see an eye doctor?
3. Every three months
4. Every six months
5. Ounce per year
6. I rarely/never checked my eye
7. Do you have a meal plan? 1. No 2. Yes
8. How many times a week do you examine your feet?
9. Once a day,
10. Once a week
11. Once a month?
12. Have you glucometer? 1. No 2. Yes
13. Do you test your blood sugar? 1. No 2. Yes
14. How many times per week should someone with diabetes exercise and for how long? _____________________________________
15. How many main meals do you have daily? _____________________________________
16. Last year, how many times did you visit a doctor? _______________________________
17. Do you smoke or drink alcohol? ___________________________________________
18. Have you ever participated in a diabetes education class? __________________________

**Thank you for your cooperation and your availability**
